# Actinobacteria Emerge as Novel Dominant Soil Bacterial Taxa in Long-Term Post-Fire Recovery of Taiga Forests

**DOI:** 10.3390/microorganisms13061262

**Published:** 2025-05-29

**Authors:** Siyu Jiang, Huijiao Qu, Zhichao Cheng, Xiaoyu Fu, Libin Yang, Jia Zhou

**Affiliations:** 1 School of Geographical Sciences, Harbin Normal University, Harbin 150025, China; 13363960039@163.com (S.J.); 15045728093@163.com (H.Q.); 2 Key Laboratory of Biodiversity, Institute of Natural Resources and Ecology, Heilongjiang Academy of Sciences, Harbin 150040, China; chengzc928@163.com (Z.C.); 18646583130@163.com (X.F.); 3 Heilongjiang Huzhong National Nature Reserve, Huzhong 165038, China

**Keywords:** soil bacteria, taiga forests, long-term post-fire recovery, diversity, dominant species

## Abstract

The long-term post-fire recovery phase is a critical stage for forest ecosystems to progress toward regeneration and mature succession. During this process, soil bacteria exhibit greater environmental adaptability, rapidly driving nutrient cycling and facilitating vegetation restoration. This study investigated the community structure and diversity of soil bacteria during long-term recovery after forest fires in the cold temperate zone, focusing on soils from the 2000 fires in Daxing’anling. Soil samples were classified into Low (L), Moderate (M), and High (H) fire damage intensity, with bacterial community composition and diversity analyzed using Illumina sequencing technology. After long-term fire recovery, the contents of soil organic carbon, black carbon, total nitrogen, alkaline nitrogen, available phosphorus, and available potassium were significantly higher elevated (*p* < 0.05), and water content was significantly lower, compared with that in the control check (CK) group. Soil urease, fluorescein diacetate, soil acid phosphatase, and soil dehydrogenase activities were significantly higher, and soil sucrase activity was significantly lower in H. There was a significant difference in the Alpha diversity index among the groups. Compared with CK, the Shannon index was significantly increased (*p* < 0.05) in L, while both Chao1 and Shannon indices were significantly decreased (*p* < 0.05) in M and significantly higher in H than CK. The results of the PCoA showed that there was a significant difference in the Beta diversity of the bacterial community among the groups (R^2^ = 0.60 *p* = 0.001). The dominant bacteria groups were Proteobacteria and Acidobacteriota, while Actinobacteria became the new dominant group during the long-term post-fire recovery. AP, WC, DOC, MBC, S-DHA, and S-SC were significantly and positively correlated with soil bacterial diversity (*p* < 0.05). The results of the co-occurrence network analysis showed that all groups were dominated by symbiotic relationships, with M having the highest network complexity and strongest competitive effects. This study found that the physicochemical properties of soils recovered over a long period of time after fire returned to or exceeded the unfired forest condition. The Actinobacteria phylum became a new dominant bacterial group, with stronger network complexity and competition, in the process of forest recovery after moderate fire.

## 1. Introduction

Fire, a natural disturbance in forest ecosystems, is affected by climate change, leading to a significant increase in the frequency of fires worldwide [1]. Forest fires in China exhibit distinct regional variations in their geographical distribution. Compared to other climatic zones, cold-temperature regions experience fewer fire incidents but suffer from more extensive burned areas, resulting in severe vegetation loss and soil exposure [2]. Forest fires exert long-term and complex impacts on forest ecosystems. On one hand, the intense heat generated during combustion can directly cause mortality or damage to surface-dwelling flora, fauna, and soil organisms. On the other hand, fires indirectly induce significant alterations in vegetation community composition and biodiversity, as well as in forest ecosystem structure and function, by modifying soil physicochemical properties, enzyme activities, and soil biological processes [3,4]. Fires have both ecological and destructive properties, causing ecosystem damage in the short term, but in the long term, they can promote plant community succession, improve soil nutrient cycling, and promote ecosystem self-regulation and recovery [5]. Previous studies have shown that after a prolonged recovery period, an increase in vegetation roots and litter can facilitate the accumulation of organic matter and nitrogen, thereby enabling the soil carbon and nitrogen content to progressively recuperate to levels that may even exceed unfired levels [6]. Furthermore, the substantial input of organic matter provides enriched nutrient substrates that facilitate the recovery and proliferation of soil microbial communities, resulting in a marked increase in microbial biomass. This enhancement consequently stimulates soil enzyme activities [7], which directly affects nutrient availability and subsequent plant growth.

In the long-term restoration of forest ecosystems, soil bacterial communities play an indispensable role through their unique biological characteristics and ecological functions. As core components of soil microbial communities, these communities exhibit short generation cycles and broad ecological niches, allowing for the rapid reassembly of community structure and functional recovery during vegetation restoration and soil environment improvement [8]. It is important for maintaining the structure and function of forest ecosystems by participating in key ecological processes such as organic matter decomposition, nutrient cycling, and maintenance of soil structure [9,10,11]. Despite these critical functions, due to the complex interplay of fire intensity, soil habitat quality, and recovery timescales, significant discrepancies persist in our current understanding of post-fire soil bacterial community dynamics. For instance, Dai et al. [12] and Sun et al. [13] found no significant differences in soil bacterial diversity and community structure between long-term post-fire recovery sites and unburned control areas. However, Hart et al. [14] and Pressler et al. [15] demonstrated that high-intensity fires may cause irreversible bacterial community shifts, with recovery patterns exhibiting distinct ecosystem dependence [16]. This ecosystem dependence is particularly evident in cold-temperature ecosystems. For example, in subalpine forests, the rapid depletion of soil organic matter triggers persistent compositional shifts in bacterial community composition that fail to return to pre-fire states even after extended recovery periods [17]. Conversely, in boreal forests, high-intensity fires cause more severe community disruption, with recovery rates being simultaneously regulated by multiple interacting factors, including soil microenvironments and vegetation succession [18,19]. Our current understanding of these differential recovery mechanisms remains limited.

The Daxing’anling forest region, a crucial cold-temperature coniferous ecosystem in northern China, is dominated by *Larix gmelinii*-based forest communities that exhibit high sensitivity to climate change. This area serves as both a sensitive indicator of global change and a fire-prone region with frequent forest fire disturbances in China. In this study, the long-term dynamics of post-fire recovery in cold-temperature larch forests were selected as the object of study. Through the integration of 16S rRNA gene amplicon sequencing and multivariate environmental factor analysis of environmental factors, the following research questions were explored: First, we examined the alterations in soil physicochemical properties following extended periods of restoration. Second, we investigated the response patterns of bacterial community structure and diversity and evaluated their associations with key environmental drivers. Third, we constructed soil bacterial co-occurrence networks to elucidate the enduring effects of fires on bacterial interaction dynamics. The results of the study provide fundamental information for elucidating the laws and mechanisms of post-fire forest and ecosystem restoration and succession. Moreover, this study offers theoretical support and practical guidance for the ecological restoration and sustainable management of burned forest land in cold-temperature regions.

## 2. Materials and Methods

### 2.1. Study Area

**Figure 1:**
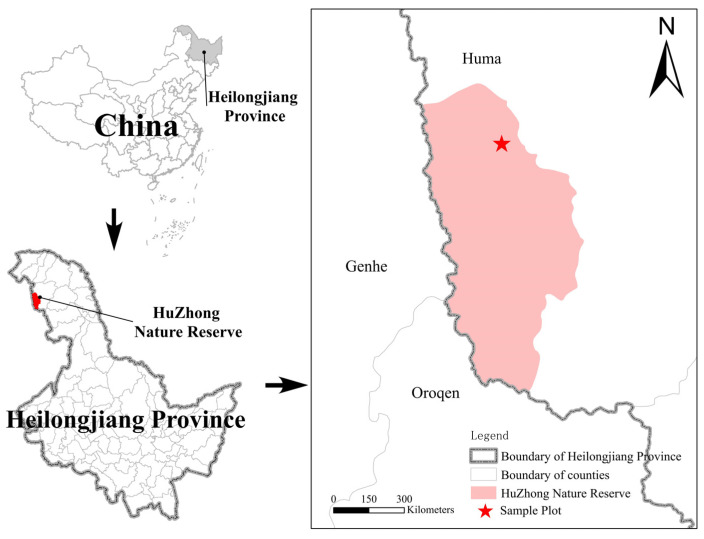
The asterisk indicates the study site in Heilongjiang Province and China. The map was generated in QGIS 3.34, using WGS 1984 coordinates, with administrative boundaries and sampling points marked.

The study area is located in Huzhong National Nature Reserve, Heilongjiang, China, with the geographical coordinates of 122°42′14″–123°18′05″ E, 51°17′42″–51°56′31″ N, as shown in Figure 1. The study area lies within the cold-temperature continental monsoon climate zone. The altitude ranges mostly between 800 and 1200 m. The region has an average annual temperature of −4 °C, an average annual precipitation of 458.3 mm, and an average annual evaporation of 911 mm. The main trees in the reserve are *Larix gmelinii*, *Pinus sylvestris* var, *mongholica Litv*, *Betula platyphylla* Suk, and *Populus davidiana* Dode; the main shrubs are *Rhododendron dauricum* L., *Ledumpalustre* L., and *Vaccinium uliginosum* L.; and the main herbs are *Maianthemum bifolium* and *Deyeuxia angustifolia* [20].

### 2.2. Sample Plots and Sample Collection

To study the long-term recovery of soil bacteria from forest fires, as shown in Table 1, based on the time series (short, medium, and long term), the forest land in the reserve burnt by the 2000 fire was selected as the long-term recovery study area [21,22]. In July 2024, based on the fire intensity classification criteria [23,24], as shown in Table 2, sample plots were set up in low (L), moderate (M), and high (H) fire areas, and a non-fire area with elevation, vegetation composition, and environmental conditions similar to those of the fire area was selected near the fire site as a control check (CK). The area of each sample plot was 20 m × 20 m, and seven replicates were set up in each group, totaling 28 experimental plots. Soil samples were collected from the 0–20 cm layer after the removal of the litter and humus layers [25]. The samples were transported back to the laboratory in an insulated box and passed through a 2 mm sieve, and small stones and plant roots were removed. One portion of the soil samples was utilized for the assessment of soil physicochemical properties and enzyme activities, while the other portion was employed for the extraction of microbial DNA.

### 2.3. Determination of Soil Physicochemical Properties and Enzyme Activities

The soil water content (WC) was determined using the drying method [26], soil pH (pH) was determined using a soil–water ratio of 1:2.5 [27], microbial biomass carbon (MBC) was quantified employing the chloroform fumigation–extraction method [28], and total nitrogen (TN) and soil organic carbon (SOC) were measured by the fully automated carbon and nitrogen analyzer Elementarvario ELIII (Elementar Analysensysteme GmbH, Langenselbold, Germany). Dissolved organic carbon (DOC) was determined by the high-temperature catalytic oxidation (HTCO) method, black carbon (BC) was determined by thermochemical oxidation with the removal of organic carbon interference, active potassium (AK) was determined by flame photometry [29], alkaline nitrogen (AN) was determined by alkaline distillation using sodium hydroxide (NaOH), and available phosphorus (AP) was determined by the sodium bicarbonate (NaHCO_3_) extraction colorimetric method [30]. Soil urease (S-UE) activity was measured using the indophenol blue method with urea as the substrate, quantifying NH_4_^+^-N absorbance after 72 h of incubation [31]. Soil sucrase (S-SC) activity was determined by the 3, 5–dinitrosalicylic acid colorimetric method, measuring reducing sugars from sucrose after 24 h at 37 °C [32]. Soil dehydrogenase (S-DHA) activity was assessed via TTC reduction, spectrophotometrically quantifying TPF after 24 h of anaerobic incubation [33]. Soil acid protease (S-ACPT) activity was analyzed using ninhydrin, measuring the amino acids from casein after 2 h at 37 °C. Soil fluorescein diacetate hydrolase (FDA) activity was determined by fluorescein release from fluorescein diacetate after 1 h at 37 °C [34].

### 2.4. Extraction and Sequencing of Bacterial DNA

Total DNA of the microbial communities was extracted according to the instructions of the E.Z.N.A.^®^ Soil DNA Kit (Omega Bio-tek, Norcross, GA, USA), the purity and concentration of the DNA were then checked by 1% agarose gel electrophoresis, the appropriate amount of sample was collected in a centrifuge tube, and the sample was diluted with sterile water to 1 ng/µL. Samples were tested against the microbial community using 341F (5′-CCTAYGGGRBGCASCAG-3′) and 806R (5′-GGACTACNNGGGTATCTAAT-3′) primers for PCR amplification of the V3-V4 variable region, and the amplification procedure was as follows: the PCR products were pre-denatured at 98 °C, for 1 min, with 30 cycles including (98 °C, 10 s; 50 °C, 30 s; 72 °C, 30 s); 72 °C, 5 min. Finally, the PCR products were aliquoted according to the PCR product concentration using a PCR instrument (Bio-rad T100 Gradient PCR Instrument Bio-Rad Laboratories, Hercules, CA, USA), and the products were purified by agarose electrophoresis using a 1xTAE agarose gel at 2% concentration after sufficient mixing, and the target bands were recovered using the Universal DNA Purification and Recovery Kit (TianGen Biotech, Beijing, China). The library was constructed using the NEB Next^®^ Ultra DNA Library Prep Kit (New England Biolabs (NEB), Ipswich, MA, USA), and the constructed library was detected and quantified by Q-PCR using Agilent 5400. After the library was qualified, it was sequenced using the Illumina sequencing platform (Illumina, San Diego, CA, USA). The raw data were uploaded to the NCBI SRA database (sequence number: PRJNA1162707).

Quality control of the raw sequenced sequences was performed using fastp software [35,36] (https://github.com/OpenGene/fastp, version 0.20.0, accessed on 6 September 2024), followed by splicing using FLASH software (http://www.cbcb.umd.edu/software/flash; version 1.2.7, accessed on 6 September 2024). The ASVs were obtained based on noise reduction using the DADA2 plug-in [37,38,39] in the QIIME 2 (version 2023.9)process, and the ASVs were analyzed taxonomically for species using the Naive Bayes classifier in Qiime2. The diversity cloud analysis platform (QIIME 2 process) was used for subsequent data analysis through Shenzhen Microcommon Technology Group Co (https://www.bioincloud.tech/, accessed on 10 September 2024).

### 2.5. Data Analysis

The raw data were processed according to the sequences, while Chao1 and Shannon indices were used to characterize the richness and alpha diversity of microbial communities. Beta diversity was calculated based on the Bray–Curtis distance, which was used to assess the differences in microbial community structure between samples and is presented in PCoA plots. We also considered the dominant bacterial community and rare species based on relative abundance of ASVs, combined with previous methods to classify conditionally abundant and rare taxa [40,41]. Data were organized and statistically analyzed using Excel 2021 and SPSS 22.0 software, and significant differences in soil physicochemical properties and enzyme activities, bacterial diversity indices, and relative abundance of bacterial communities were analyzed by one-way analysis of variance (ANOVA). The Mantel test and Spearman’s analysis were used to reveal potential associations between microbial communities and related environmental factors. The “igraph” and “psych” packages of the R-3.3.1 were used to construct co-occurrence networks of microbial communities, and Gephi.10.1 was used to visualize and compute the topological properties of the co-occurrence network and assess the complexity and stability of the microbial network.

## 3. Results

### 3.1. Changes and Differences in Soil Physicochemical Properties and Enzyme Activities After Long-Term Restoration of Burned Forest Land

As shown in Table 3, after the long-term restoration of fire-burned cold-temperature larch forests, the soil moisture content was significantly lower than CK (*p* < 0.05), but the contents of SOC, BC, TN, AN, AP, and AK were significantly higher than CK. Notably, the content of AK increased significantly with increasing fire intensity. Soil pH was significantly higher in L and M than CK but significantly lower in H. Overall, pH decreased significantly with increasing fire intensity. Soil MBC and DOC contents were significantly higher in L and H compared to the CK but significantly lower in M.

The activity of S-SC was significantly lower than CK (*p* < 0.05), while the activities of S-UE, FDA, and S-DHA were significantly higher than CK, with S-UE increasing with fire intensity. Notably, in H, the activities of S-UE, FDA, S-ACPT, and S-DHA were significantly higher than other groups.

### 3.2. Characterizing the RESToration of Soil Microbial Community Diversity

**Figure 2:**
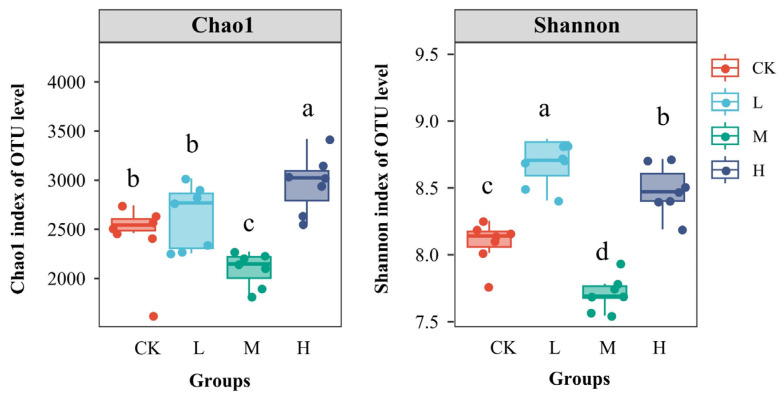
Alpha diversity of soil bacterial communities. Different letters within a row indicate significant differences (*p* < 0.05, ANOVA) among the different intensities of fire in this study.

As shown in Figure 2, there was no significant change in the Chao1 index and a significant increase in the Shannon index in L compared to CK (*p* < 0.05). Furthermore, the Chao1 and Shannon indices of soil bacteria in M were significantly lower than those in the other groups. In contrast, both indices in H were significantly higher than those in CK. The study revealed significant differences in the diversity and abundance of soil bacterial communities following the prolonged restoration of burned forest land subjected to fire. The alpha diversity of forest soil bacteria after M disturbance remained lower than pre-fire levels. In contrast, after H disturbance, it not only recovered but also exceeded pre-fire levels.

**Figure 3:**
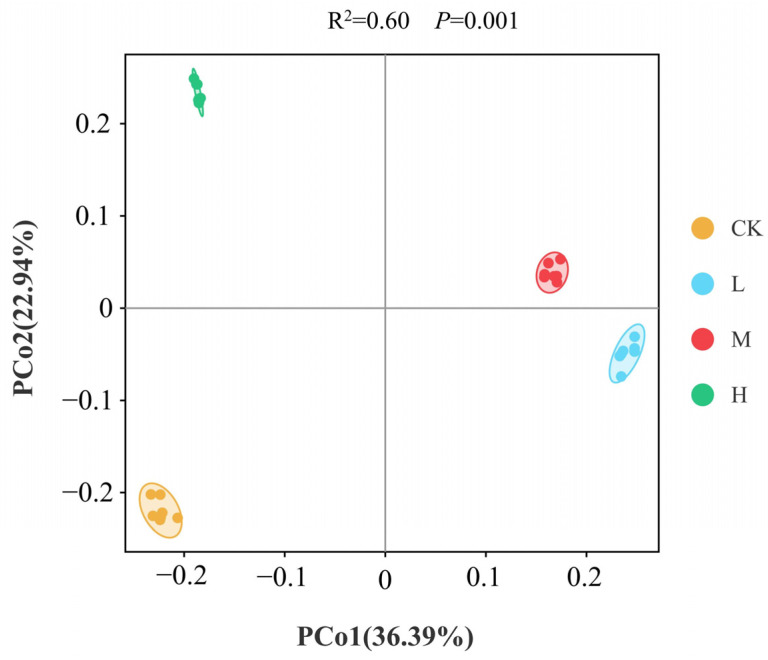
Principal Coordinate Analysis (PCoA) of bacterial communities in soil of different intensity fire sites with control (CK, no fire), low fire (L), moderate fire (M), and high fire (H). For each fire site, seven independent samples were analyzed.

Based on the Bray–Curtis distance, Principal Coordinate Analysis (PCoA) was used to analyze changes in bacterial community Beta diversity. As shown in Figure 3, the PCo1 and PCo2 axes explained 36.39% and 22.94% of the variance, respectively, with a cumulative variance of 59.33%. The bacterial community structure was clearly differentiated among groups. The ANOSIM test results indicated significant differences in bacterial community Beta diversity between groups (R^2^ = 0.60, *p* = 0.001). The results indicated that even after long-term recovery following fire disturbance, the bacterial Beta diversity of forest soil remained significantly different from pre-fire levels.

### 3.3. Microbial Community Composition and Restoration Characteristics of Community Types

**Figure 4:**
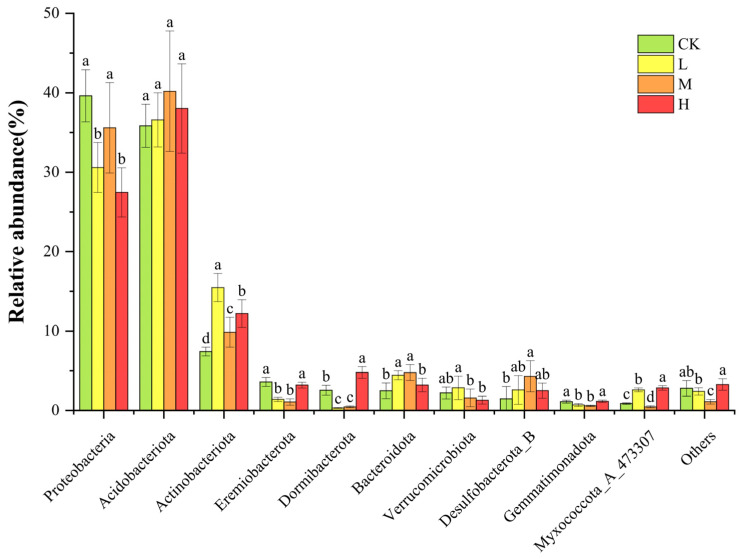
Soil bacterial community composition at the phylum level. Different letters above bars indicate significant differences (*p* < 0.05, ANOVA) among the different intensities of fire in this study.

As shown in Figure 4, the predominant soil bacterial phyla were Proteobacteria (27.47% to 39.63%) and Acidobacteriota (35.85% to 40.20%). Compared with the CK, the relative abundance of Proteobacteria was significantly lower (*p* < 0.05) in L and H. In contrast, the relative abundance of Acidobacteriota showed no significant differences among groups. The relative abundance of Actinobacteria (7.43% to 15.48%) was significantly higher (*p* < 0.05) in the fire groups and became the dominant phylum. In addition, the relative abundance of Eremiobacterota, Dormibacterota, and Gemmatimonadota was significantly lower (*p* < 0.05), and Bacteroidota’s relative abundance was significantly higher (*p* < 0.05) in groups L and M compared to CK. In M, Desulfobacterota_B increased significantly (*p* < 0.05), while Myxococcota_A_473307 decreased. Notably, Myxococcota_A_473307 significantly increased in L and H (*p* < 0.05) compared to CK, whereas Verrucomicrobiota showed no significant changes among groups.

As shown in Table 4, the proportion of dominant ASV taxa was less than 1% compared to the control groups, while rare taxa accounted for approximately 90% of the total. Concerning the number of rare ASVs, the number of rare species (RT) ASVs was ranked as L > H > CK > M, and the proportion of rare species ASVs was ranked as L > CK > H > M. Furthermore, the number and proportion of dominant species (DT) ASVs in moderately burned forests was higher than that of other groups.

### 3.4. Differences Correlation Analysis

#### 3.4.1. Microbial Community Composition and Diversity Correlated with Soil Physicochemical Properties and Enzyme Activities

**Figure 5:**
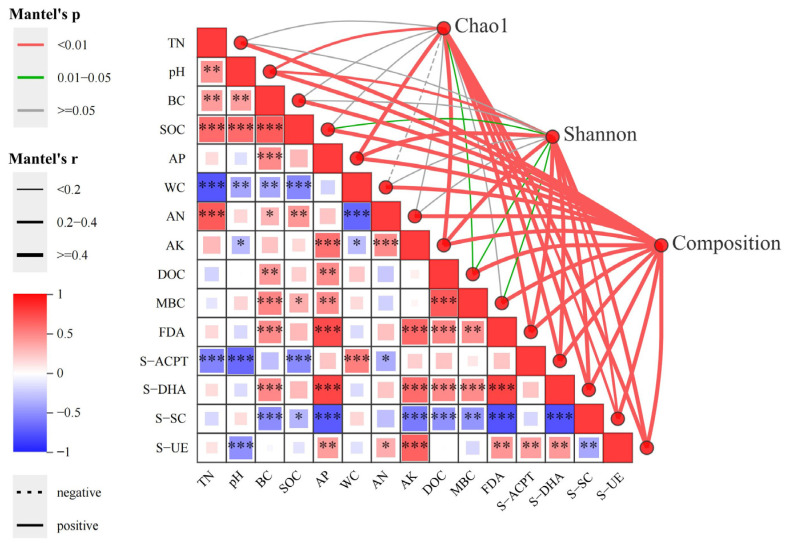
Mantel test of soil bacterial community with soil physicochemical properties and enzyme activities. Left: Mantel test results showing correlations between soil physicochemical properties (TN, pH, etc.) and enzyme activities (FDA, S-DHA, etc.). Color gradient indicates Mantel’s r values, and significance is indicated. Right: α-diversity indices (Chao1, Shannon) and community composition correlations with environmental factors. Significance is indicated as * for 0.01 < *p* ≤ 0.05, ** for 0.001 < *p* ≤ 0.01, *** for *p* ≤ 0.001.

As shown in Figure 5, the Mantel test revealed significant positive correlations between the Chao 1 index and AP, WC, DOC, and MBC (*p* < 0.01). Similarly, the Shannon index showed a highly significant and positive correlation with pH, AP, WC, AN, DOC, and MBC. Among these factors, AP, WC, DOC, and MBC were identified as the main factors influencing the alpha diversity of soil bacteria. Notably, WC showed a significant negative correlation with TN, pH, BC, SOC, and AN. Additionally, other factors exhibited significant positive correlations. The bacterial community composition showed highly significant positive correlations with all physical and chemical factors.

The results showed that both Chao 1 and Shannon indices were significantly positively correlated with S-DHA and S-SC (*p* < 0.01), which were the main factors influencing soil bacterial alpha diversity. Bacterial community composition had a significant positive correlation with enzyme activities, while S-SC was highly significantly and negatively correlated with FDA, S-DHA, and S-UE.

#### 3.4.2. Species Composition Correlates with Soil Physicochemical Properties and Enzyme Activities

**Figure 6:**
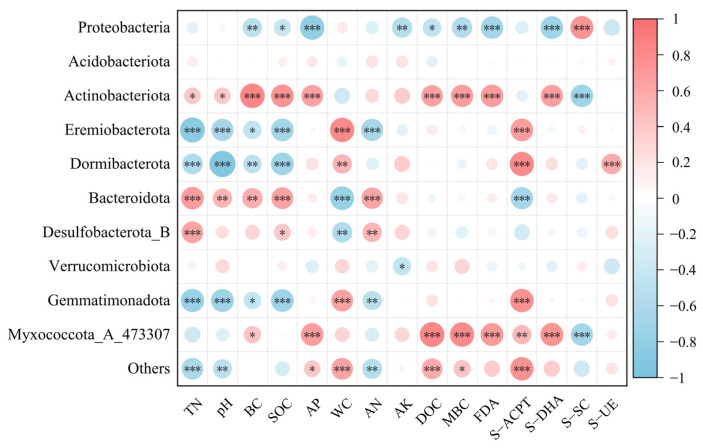
Heatmap of Spearman correlation between soil bacterial communities and physicochemical properties and enzyme activities. Red identifies positive and blue identifies negative correlations, with darker colors for stronger correlations. Significance is indicated as * for 0.01 < *p* ≤ 0.05, ** for 0.001 < *p* ≤ 0.01, *** for *p* ≤ 0.001.

Soil bacterial phyla were selected for correlation analysis with soil physicochemical properties and enzyme activities, and the results are shown in Figure 6, where BC, SOC, AP, AK, DOC, and MBC were significantly negatively correlated with Proteobacteria (*p* < 0.05) and significantly positively correlated with Actinobacteriota. TN, pH, BC, and SOC were significantly negatively correlated with Eremiobacterota, Dormibacterota, and Gemmatimonadota and significantly positively correlated with Bacteroidota. WC was significantly positively correlated with Eremiobacterota, Dormibacterota, and Gemmatimonadota and significantly negatively correlated with Bacteroidota and Desulfobacterota_B. In addition, TN, SOC, and AN were significantly positively correlated with Desulfobacterota_B. AK was significantly negatively correlated with Verrucomicrobiota. BC, AP, DOC, and MBC were significantly and positively correlated with Myxococcota_A_473307. AP, WC, DOC, and MBC were significantly and positively correlated with Others, and TN, pH, and AN were significantly and negatively correlated with Others.

FDA and S-DHA were significantly negatively correlated with Proteobacteria (*p* < 0.05) and highly significantly positively correlated with Actinobacteria and Myxococcota_A_473307. S-ACPT was highly significantly positively correlated with Eremiobacterota, Dormibacterota, Gemmatimonadota, Myxococcota_A_473307, and Others and highly significantly negatively correlated with Bacteroidota. S-SC was significantly positively correlated with Proteobacteria and highly significantly negatively correlated with Actinobacteria and Myxococcota_A_473307. S-UE was highly significantly positively correlated with Dormibacterota.

### 3.5. Co-Occurrence Network Analysis of Soil Microbial Communities After Long-Term Restoration of Burned Forests

**Figure 7:**
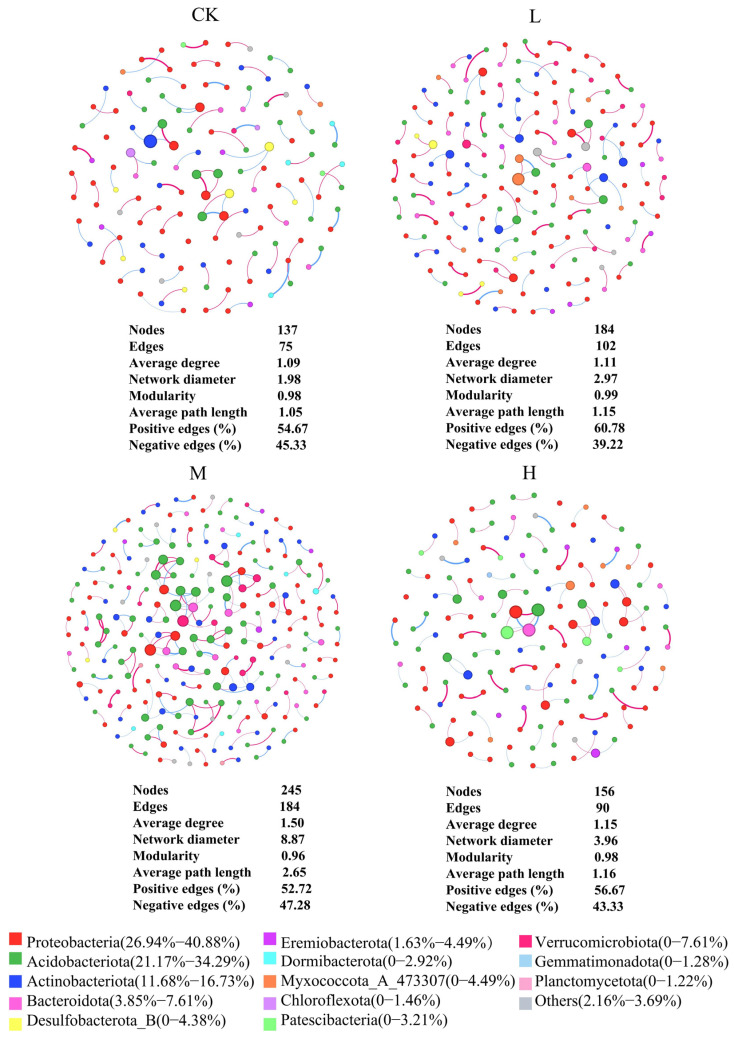
Co-occurrence network analysis and topological characteristics of soil bacterial communities. (1) Significant correlations (Spearman’s r > 0.6, *p* < 0.05) between taxa, with node size proportional to connectivity degree and color indicating bacterial phyla; (2) relative abundance distribution of network-represented phyla; (3) Topological parameters including average path length, clustering coefficient, and modularity.

As shown in Figure 7, Proteobacteria (26.94% to 40.88%), Acidobacteriota (21.17% to 34.29%), and Actinobacteria (11.68% to 16.73%) were the dominant phyla in the co-occurrence network of burned forest land. Among these phyla, the relative abundance of Proteobacteria in the fire-disturbed groups was lower than CK, while Acidobacteriota and Actinobacteria showed higher relative abundances compared to CK. Additionally, the relative abundances of non-dominant phyla varied significantly among different groups. The results indicated that the original dominant taxa in the forest soil remained the main components of the soil bacterial network even after long-term recovery from fire disturbance. Meanwhile, low-abundance taxa also played a significant role in maintaining the structure of the soil bacterial community.

From Figure 7, it also can be seen that the values of the number of nodes, number of edges, average degree, and network diameter in the fire groups were higher than CK. This indicates that the complexity of the soil bacterial network increased after long-term recovery from fire. Among the groups, M showed the highest values for the number of nodes, number of edges, average degree, average path length, and network diameter, along with the lowest modularity. This suggests that the soil bacterial network complexity was highest in M following long-term recovery. In addition, the proportion of positive correlations among nodes in the bacterial co-occurrence network exceeded that of the negative correlations in all groups. This indicates that mutualistic relationships, particularly symbiosis, were more prevalent than competition. L exhibited the highest proportion of positive correlations, while M showed the highest proportion of negative correlations. This suggests that symbiotic relationships were dominant overall, but competitive interactions were relatively stronger in M.

## 4. Discussion

### 4.1. Effects on Soil Physicochemical Properties and Enzyme Activities After Long-Term Restoration of Burned Forest Land

It has been demonstrated that the physical structure, chemical properties, and microbial activity of forested soils undergo changes following long-term recovery from fire [8,42]. In this study, a significant decrease in soil moisture content was observed after prolonged recovery, which may be attributed to delayed vegetation regeneration in cold-temperature fire-affected forests. In these sites, tree diameter at breast height and canopy closure remained significantly lower than pre-fire levels, resulting in expanded canopy gaps and increased soil exposure. The expansion of forest gaps allows more solar radiation to reach the ground, increasing soil evaporation and ultimately decreasing soil moisture [19]. The study revealed that the soil MBC and DOC contents in L and H were significantly higher than control groups. Possible explanations for this phenomenon include the fact that it is due to the high-temperature effect of the fire, which promoted the partial mineralization of soil organic matter, generating substantial readily decomposable organic carbon. The mineralized organic carbon not only provided abundant energy and substrates for microorganisms, directly stimulating their growth and increasing MBC content, but also rapidly dissolved into the soil solution as DOC due to its high water solubility [43]. As vegetation recovers after fire, it increases root exudates and litter input, providing sustained organic carbon sources. This long-term accumulation of plant-derived organic matter significantly elevates soil MBC and DOC contents [44,45]. The elevated SOC and BC content resulted directly from fire-generated black carbon particles, charred residues, and other pyrogenic organic inputs, which led to the accumulation of soil carbon pools during the restoration process [46]. Both soil TN and AN contents showed significant increases, consistent with previous findings [47,48]. This occurs primarily through thermal mineralization during combustion, which transforms soil organic N into inorganic forms. The resulting ammonium N becomes adsorbed onto negatively charged soil colloids (clay minerals and organic matter), leading to N retention in soils [43,49]. The findings revealed a substantial increase in both soil AP and AK contents, which is consistent with the conclusions of earlier studies [3,50]. This stems not only from the combustion-induced release of readily available nutrients but also from continuous nutrient mineralization through litter decomposition and microbial activity during post-fire ecological succession [51]. The results of a large number of recent studies indicate that burning leads to an increase in pH, which is consistent with the results of the low- and moderate-fire groups in this study. Due to the relatively low damage to soil organic matter caused by low and moderate burning, the mineralization of organic matter produces less acidic material and alkaline inputs dominate [42]. However, in this study, pH was significantly lower in high than in control groups, which may be attributed to the combination of continuous alkaline loss and accumulation of acids (e.g., humic acids) generated by organic matter mineralization during the 20-year restoration process, which ultimately led to increased soil acidification [52].

The enzymes present in soil during post-fire forest recovery primarily originate from soil microorganisms, plant root exudates, and the decomposition of organic residues. Therefore, soil enzyme analysis serves as a sensitive proxy for microbial metabolic activity and can help to evaluate post-fire impacts on soil quality and ecosystem functioning [53]. Following the destruction of above-ground vegetation and apomictic material in the aftermath of fire, soil enzyme activities exhibited a trend of a significant decrease and subsequent increase with the recovery time. A previous study demonstrated that the vegetation of the Daxing’anling burned forest land was stabilized only after 30 years of recovery [54], and further research still requires longer-term monitoring data. The activity of S-SC enzymes was significantly lower than that of the control group in the present study. This reduction can be primarily attributed to the thermal degradation of sensitive microbial communities, especially sucrose-dependent microorganisms, resulting in reduced S-SC activity [5]. The enzymatic activities of S-UE, FDA, S-ACPT, and S-DHA in fire-affected areas were significantly elevated compared to control groups. This enhancement can be attributed to fire-induced alterations in community succession patterns, leading to increased biodiversity and substrate availability. The long-term restoration process in severely burned forest land resulted in substantial accumulations of plant residues, root exudates, and organic matter, which provided diverse substrates for microbial decomposition. These conditions subsequently stimulated enzymatic activities, a phenomenon consistent with previous research findings [55,56]. In summary, the changes in soil physicochemical parameters and enzyme activities after recovery show significant variation across fire-affected forests due to the combined effects of fire intensity, recovery time, vegetation type, below-ground microbial communities, and soil heterogeneity.

### 4.2. Impacts on Soil Bacterial Diversity After Long-Term Restoration of Burned Forest Land

Microbial diversity captures post-disturbance microbial community reorganization and their functional resilience, serving as a key bioindicator for assessing soil ecosystem recovery [57]. The results of the Mantel test in this study revealed that AP, WC, DOC, and MBC were significantly and positively correlated with bacterial alpha diversity indices. The elevated AP content not only directly enhanced microbial metabolic activity but also indirectly promoted microbial diversity by increasing the availability of labile carbon substrates, particularly amino acids, amines, and phenolic compounds [58,59]. Variations in DOC content reflect changes in readily available carbon sources for microorganisms under different fire intensities during long-term recovery. These DOC dynamics have been shown to either stimulate or suppress soil bacterial growth and metabolic activities, consequently affecting bacterial diversity [60,61]. Furthermore, the reduced WC content may have enhanced soil aeration, thereby favoring the diffusion of oxygen through the soil and increasing soil oxygen content. This environmental shift has the potential to promote the growth and metabolic activities of aerobic microorganisms, which, in turn, can influence the composition and diversity of the bacterial community [13,43]. The elevated S-DHA activity, as a key microbial intracellular enzyme, indicates enhanced microbial metabolic activity and functional diversity, reflecting the active state of microbial communities [62]. Conversely, the reduced S-SC activity likely facilitates ecological niche differentiation, promoting bacterial taxa coexistence and thereby maintaining higher diversity levels [62]. These microbial responses may be attributed to the gradual improvement of soil environmental conditions during the restoration process, which creates favorable habitats for microbial community recovery and diversity maintenance [13].

Previous research has demonstrated that alpha diversity fluctuations primarily result from the disappearance or enrichment of rare species at large scales. In this study, we divided bacterial taxa into groups based on ASV abundance and found that changes in alpha diversity were mainly influenced by the following factors: Firstly, rare species, characterized by their low abundance and stringent ecological niche requirements [63], are particularly vulnerable to environmental disturbances, often resulting in substantial population declines [64]. During post-fire restoration, alterations in vegetation conditions and soil environments can trigger ecological niche reconfiguration of rare species, ultimately leading to fluctuations in alpha diversity. AP, DOC, and WC were found to be significantly and positively correlated with Chao1 and Shannon in this study. Secondly, rare species exhibit lower competitive ability compared to dominant species. The increase in dominant species or intensified community competition may lead to the exclusion of rare species, consequently reducing community diversity [65,66], and vice versa. Furthermore, as shown in Figure 7, the moderate-fire groups demonstrated the highest number of dominant species and the lowest rare species abundance after long-term restoration. This group also showed the highest proportion of negative correlations in the ecological network, indicating the strongest competitive interactions in moderate groups, which subsequently resulted in decreased alpha diversity. These findings strongly support our second proposed mechanism.

### 4.3. Relationship Between Soil Microbial Community Characteristics and Soil Physicochemical Properties and Enzyme Activities

The distribution of microbial communities demonstrates high sensitivity to variations in soil environmental factors. These factors play a crucial role in shaping microbial community structures during long-term post-fire forest restoration [67]. In this study, Proteobacteria, Acidobacteria, and Actinobacteria emerged as the dominant bacterial phyla across all groups in long-term post-fire forest restoration sites. These phyla are characterized by broad ecological amplitudes [68] and show strong adaptability to environmental changes [69,70]. Notably, Proteobacteria and Bacteroidetes, recognized as typical r-strategists, showed a decrease in the relative abundance of Proteobacteria, which aligns with previous research findings [71]. Firstly, the altered environmental conditions became unfavorable for the growth of oligotrophic groups within Proteobacteria, consequently reducing their relative abundance. This explains the significant negative correlation observed between Proteobacteria and soil nutrients in our study [72]. Secondly, although Proteobacteria can utilize readily available carbon sources for rapid growth, the decreased S-SC activity inhibits the decomposition of soil glucose and root exudates, ultimately reducing Proteobacteria’s relative abundance due to limited carbon availability [73]. As copiotrophic taxa, Bacteroidetes benefit from elevated soil nutrient levels during long-term restoration, resulting in increased relative abundance of this bacterial phylum. Both Actinobacteria and Acidobacteria, recognized as typical k-strategists, play crucial roles in organic matter decomposition processes [74]. Acidobacteria, classified as acidophilic bacteria, typically exhibit higher abundance in acidic environments [75,76]. In this study, Acidobacteria showed no significant differences in relative abundance across groups and remained unaffected by pH variations. This phenomenon may be attributed to their strong acid tolerance, as the observed pH fluctuations fell within their adaptive range [77]. Notably, Actinobacteria emerged as one of the dominant phyla in long-term recovered fire-affected soils, which may be attributed to their exceptional carbon-cycling capabilities and efficient nutrient competition strategies, ultimately enabling them to establish dominance within the soil microbial community [78]. Our analysis of relative abundance differences between r-strategists and k-strategists revealed a significantly lower abundance of r-strategists in burned sites compared to control groups. In contrast, k-strategists maintained over 50% relative abundance in burned sites, significantly exceeding control groups. Previous studies have documented the rapid proliferation of r-strategists immediately after fire events, followed by a gradual decline in their competitive advantage over time, which aligns with our findings [13,79]. These results indicate that k-strategists become dominant during long-term post-fire recovery, with oligotrophic functional groups prevailing in the soil bacterial community.

### 4.4. Co-Occurrence Network Analysis of Soil Microbial Communities

Microbial co-occurrence network analysis effectively reveals response mechanisms of microbial interactions to environmental disturbances. Significant differences were observed in both topological properties and network structures of microbial communities across varying fire intensities. In this study, Proteobacteria, Actinobacteria, and Acidobacteria are identified as the keystone phyla across all microbial networks, playing crucial roles in ecological network formation. Notably, the relative abundance of Actinobacteria and Acidobacteria phyla in the network was similar to the results of community composition at the phylum level after long-term recovery compared to the control groups. As typical k-strategists, they facilitate environmental adaptation through improved survival rates and competitive capacity, consequently maintaining population stability and high saturation density [80]. The decreased relative abundance of Proteobacteria in the network likely stems from other microbial taxa’s successful adaptation to post-fire conditions, gaining competitive advantages in resource utilization and microbial interactions and consequently reducing Proteobacteria’s network significance. We detected a fundamental transition in microbial life-history strategies between undisturbed and fire-impacted ecosystems: control soils were characterized by r-strategist dominance, whereas post-fire communities exhibited k-strategist predominance, corresponding to their respective community structures. Crucially, k-strategists served as key stabilizers in post-fire microbial networks. Their self-regulatory capacity, mediated through prolonged environmental feedback, drove measurable increases in both the structural complexity and functional stability of the soil microbiome [81].

This study revealed the profound impacts of fire on soil microbial interactions and ecological functions through a comparative analysis of co-occurrence network topological properties between burned and control sites. The microbial co-occurrence network in fire-affected soils exhibited significantly higher topological parameters (including node number, edge count, and average degree) compared to control groups, with the most pronounced effects observed in moderate groups, suggesting that the bacterial communities in the burned forest have stronger interactions with each other and that the microbial co-occurrence network is more complex, implying a more efficient transfer of soil nutrient resources [42,82]. Notably, these network characteristics may be attributable to moderate groups, which optimally enhance microbial interactions while preventing severe soil environmental damage. In the context of microbial community interactions, the prevalence of positive correlations within the bacterial co-occurrence network was found to exceed that of negative correlations. This observation suggests that bacterial communities predominantly engage in symbiotic interactions rather than competitive ones [83,84]. Specifically, low groups exhibited the highest proportion of positive correlations, indicating that its bacterial community was predominantly characterized by symbiotic relationships. The environmental impact of L was found to be minimal, as it retained a substantial amount of organic matter and maintained ecological niche diversity. This, in turn, led to the proliferation of abundant resources for microorganisms, thereby fostering the establishment of mutually beneficial symbiotic relationships [85]. In contrast, moderate groups showed a higher proportion of negative correlations, indicating more pronounced competitive interactions within their bacterial community. Moderate-fire groups caused greater environmental damage, resulting in resource depletion and niche compression, which intensified microbial competition for limited resources [86]. High groups are the most environmentally destructive, significantly reducing the diversity and abundance of microbial communities, with only a few tolerant species surviving, leading to a simplification of microbial interactions and an equalization of network structure [8]. This simplification reduces complex symbiotic or competitive relationships and leads to a more balanced network topological parameter, which facilitates resource sharing and avoids excessive competition, thus maintaining the stability of the microbial community and the continuity of ecological functions. Furthermore, moderate competitive relationships have been demonstrated to enhance species interactions, maintain biodiversity, and improve the community’s capacity for self-regulation and recovery from external disturbances [87,88].

The comprehensive results demonstrated that the soil bacterial community in the burned forest land after prolonged recovery developed a more intricate network of mutualistic relationships compared to that in the undisturbed land. This heightened ecological complexity was reflected not only in a systematic improvement in the functional stability but also in the diversity maintenance capacity of the community. During the long-term recovery phase, the dynamics of soil environmental factors reshape the microbial community through dual roles: (1) the direct regulation of network complexity and stability and (2) indirect effects on species diversity mediated by shifts in resource availability and habitat conditions. Collectively, these changes fostered a more resilient soil microbial ecosystem. Consequently, future research on soil bacterial communities should prioritize not only species diversity assessments but also the interactions among major taxa and their potential ecosystem functional impacts. This approach provides new insights into the recovery mechanisms and ecological roles of post-fire soil microbial communities, offering a valuable theoretical framework for forest ecosystem management and restoration.

## 5. Conclusions

Significant changes were observed in soil physicochemical properties, enzyme activities, and microbial community structure after the long-term restoration of burned forest land. The main findings are as follows: (1) Soil physicochemical properties were significantly altered but showed decoupled dynamics with microbial activity, indicating the incomplete recovery of ecosystem functionality. (2) Soil bacterial diversity was primarily shaped by key environmental factors, with oligotrophic k-strategists (adapted to nutrient-limited conditions) becoming the dominant group. (3) Post-fire soils exhibited increased complexity in bacterial co-occurrence networks, with moderate fire displaying the highest network complexity and competitive intensity. Future research should investigate the long-term successional dynamics of microbial communities across varying fire intensities, as well as their regulatory roles in ecosystem functioning. These insights will provide a scientific foundation for ecological restoration and management after forest fires.

## Figures and Tables

**Table 1:** Classification of post-fire recovery stages by time interval and fire intensity.

Recovery Stage	Time Scale	Character
Short Term	0–5 years	Sharp decline in bacterial diversity, outbreaks of actinomycetes, carbon cycle dysfunction
Medium Term	5–20 years	Re-establishment of nitrogen-fixing bacterial communities and gradual recovery of bacterial mutualistic networks
Long Term	>20 years	Microbial diversity stabilizes, but functional genes still lag behind

**Table 2:** Basic situation of fire sites with different intensities. L, M, and H indicate low, moderate, and high fire intensities, respectively.

Fire Intensity	Flame Height	Victimization of Standing Timber	Severity
Low fire (L)	≤1.5 m	≤30%	Burned bark and stems, tree with green leaf cover
Moderate fire (M)	1.5–3 m	30–70%	Charred stems, trees still have green leaf cover
High fire (H)	≥3 m	≥70%	Burned canopy, no green leaf cover

**Table 3:** Physicochemical properties and enzymatic activities in soil. Data in the table are presented as mean ± standard error (n = 7). Different letters within a row indicate significant differences (*p* < 0.05, ANOVA) among the different intensities of fire in this study.

Index	CK	L	M	H
TN g/kg	1.65 ± 0.03 d	3.99 ± 0.06 b	4.47 ± 0.10 a	3.22 ± 0.02 c
pH	4.31 ± 0.01 c	4.78 ± 0.00 a	4.38 ± 0.01 b	4.20 ± 0.01 d
BC g/kg	7.07 ± 0.40 c	51.39 ± 0.88 a	33.97 ± 0.91 b	34.12 ± 1.77 b
SOC g/kg	12.00 ± 0.28 d	78.81 ± 1.21 a	52.02 ± 0.94 b	48.02 ± 0.78 c
AP mg/kg	20.46 ± 0.17 d	39.66 ± 0.24 b	29.35 ± 0.31 c	59.63 ± 0.09 a
WC %	41.14 ± 1.46 a	29.77 ± 1.51 c	18.77 ± 0.76 d	32.20 ± 3.10 b
AN mg/kg	50.93 ± 0.52 c	104.43 ± 2.60 b	143.82 ± 2.34 a	108.12 ± 7.02 b
DOC mg/kg	145.95 ± 3.76 c	282.34 ± 10.79 a	125.49 ± 2.57 d	273.30 ± 5.24 b
AK mg/kg	172.44 ± 2.41 d	267.83 ± 3.09 c	276.83 ± 2.63 b	444.96 ± 3.39 a
MBC mg/kg	368.84 ± 6.65 c	579.24 ± 13.49 a	351.59 ± 12.36 d	493.66 ± 7.00 b
FDA μmol/d	1.20 ± 0.06 c	3.93 ± 0.22 b	1.41 ± 0.09 c	13.03 ± 0.32 a
S-ACPT mg/d	1.57 ± 0.10 b	1.22 ± 0.07 c	1.20 ± 0.10 c	3.39 ± 0.25 a
S-DHA μg/d	40.55 ± 1.25 d	100.53 ± 6.11 b	62.4 ± 4.06 c	160.26 ± 7.20 a
S-SC mg/d	61.54 ± 2.98 a	44.71 ± 2.10 c	54.15 ± 0.99 b	39.85 ± 1.94 d
S-UE μg/d	168.67 ± 1.75 c	168.58 ± 9.55 c	302.39 ± 15.65 b	1088.76 ± 17.18 a

TN: total nitrogen; pH: soil pH; BC: black carbon; SOC: soil organic carbon; AP: available phosphorus; WC: soil water content; AN: alkaline nitrogen; DOC: dissolved organic carbon; AK: active potassium; MBC: microbial biomass carbon. FDA: fluorescein diacetate; S-ACPT: soil acid phosphatase; S-DHA: soil dehydrogenase; S-SC: soil sucrase; S-UE: soil urease. CK: Control Check; L: Low fire; M: Moderate fire; H: High fire.

**Table 4:** Differences in soil bacterial community types. The values in the table indicate the number and proportion of species.

Index	CK	L	M	H
AAT	10 (0.28%)	7 (0.16%)	9 (0.31%)	7 (0.18%)
CAT	6 (0.17%)	11 (0.26%)	13 (0.45%)	9 (0.23%)
MT	296 (8.27%)	301 (7.08%)	271 (9.48%)	354 (8.92%)
CRAT	0 (0.00%)	2 (0.05%)	0 (0.00%)	0 (0.00%)
CRT	1731 (48.37%)	2311 (54.35%)	1208 (42.24%)	1904 (47.96%)
ART	1536 (42.92%)	1620 (38.10%)	1359 (47.52%)	1696 (42.72%)
DT	16 (0.45%)	20 (0.47%)	22 (0.77%)	16 (0.40%)
RT	3267(91.28%)	3931 (92.45%)	2567 (89.76%)	3600 (90.68%)

Dominant species (DT) are the sum of absolutely abundant species (AAT), conditionally abundant species (CAT), and conditionally rich or rare species (CRAT); rare species (RT) are the sum of absolutely rare species (ART) and conditionally rare species (CRT); and MT are intermediate species. CK: Control Check; L: Low fire; M: Moderate fire; H: High fire.

## Data Availability

Data are contained within the article.
